# Role of Sex Hormones in the Development and Progression of Hepatitis B Virus-Associated Hepatocellular Carcinoma

**DOI:** 10.1155/2015/854530

**Published:** 2015-09-27

**Authors:** Maurizio Montella, Giovanni D'Arena, Anna Crispo, Mario Capunzo, Flavia Nocerino, Maria Grimaldi, Antonio Barbieri, Anna Maria D'Ursi, Mario Felice Tecce, Alfonso Amore, Massimiliano Galdiero, Gennaro Ciliberto, Aldo Giudice

**Affiliations:** ^1^Epidemiology Unit, National Cancer Institute of Naples “G. Pascale Foundation”, IRCCS, 80131 Naples, Italy; ^2^Department of Onco-Hematology, IRCCS, Cancer Referral Center of Basilicata, 85028 Rionero in Vulture, Italy; ^3^Department of Medicine and Surgery, University of Salerno, 84081 Fisciano, Italy; ^4^Animal Facility, National Cancer Institute of Naples “G. Pascale Foundation”, IRCCS, 80131 Naples, Italy; ^5^Department of Pharmacy, University of Salerno, 84084 Fisciano, Salerno, Italy; ^6^Department of Surgery, National Cancer Institute of Naples “G. Pascale Foundation”, IRCCS, 80131 Naples, Italy; ^7^Department of Experimental Medicine, II University of Naples, 81055 Naples, Italy; ^8^National Cancer Institute “G. Pascale Foundation”, IRCCS, 80131 Naples, Italy

## Abstract

Infection with hepatitis B virus (HBV) is a major risk factor for hepatocellular carcinoma (HCC) in developed countries. Epidemiological reports indicate that the incidence of HBV-related HCC is higher in males and postmenopausal females than other females. Increasing evidence suggests that sex hormones such as androgens and estrogens play an important role in the progression of an HBV infection and in the development of HBV-related HCC. While androgen is supposed to stimulate the androgen signaling pathway and cooperate to the increased transcription and replication of HBV genes, estrogen may play a protecting role against the progression of HBV infections and in the development of HBV-related HCC through decreasing HBV RNA transcription and inflammatory cytokines levels. Additionally, sex hormones can also affect HBV-related hepatocarcinogenesis by inducing epigenetic changes such as the regulation of mRNA levels by microRNAs (miRNAs), DNA methylation, and histone modification in liver tissue. This review describes the molecular mechanisms underlying the gender disparity in HBV-related HCC with the aim of improving the understanding of key factors underneath the sex disparity often observed in HBV infections. Furthermore, the review will propose more effective prevention strategies and treatments of HBV-derived diseases.

## 1. Introduction

Hepatocellular carcinoma (HCC) is one of the primary malignant liver tumors more common and is a serious worldwide public health problem [1, 2]. In developed countries, some infectious diseases and mainly infections due to hepatitis viruses (both hepatitis B virus (HBV) and hepatitis C virus (HCV)) are a major risk factor for the development of hepatocellular carcinoma [1, 3]. Many other risk factors are associated with the presence of liver cirrhosis which is a premalignant condition of HCC; in fact, 80–90% of HCC cases develop as a result of liver cirrhosis. These predisposing factors can be identified with alcohol use, diabetes, obesity, intake of aflatoxin contaminated-food, nonfatty liver disease (NAFLD), and genetic diseases such as hemochromatosis and *α*-antitrypsin [1, 4, 5]. Despite the fact that cirrhosis is the main etiological factor contributing to the hepatocarcinogenesis process, a large number of patients without cirrhosis develop hepatocellular carcinoma, suggesting that the disease process involves oncogenic events and virus-related factors [6]. Interestingly, marked epidemiological variations, which reflect geographic regions, but also differences on the genetic basis of ethnicity and race have been observed and described in the literature [5, 7–10]. Notably, hepatitis B virus (HBV) X protein (HBx) has been reported to play an important role in the development of HCC during HBV infection [11]. HBx protein regulates transcription of several cellular and viral genes by interacting with cellular proteins and/or components of signal transduction pathways [12–16]. The interaction of HBx with these proteins could be involved in neoplastic transformation of hepatocytes [12–16]. Another important aspect is that sex hormones can cause the chronic release of inflammatory cytokines in the hepatocellular microenvironment [17–19] and induce epigenetic changes such as the regulation of mRNA levels by microRNAs (miRNAs), DNA methylation, and histone modification [20–23]. One remarkable clinical feature of HBV-associated HCC is that its incidence strikes many more men and postmenopausal females than other females and that it may be a type of hormone-responsive tumor [22, 24–26]. This epidemiologic observation makes the male gender an important risk factor for HBV-related hepatocarcinogenesis. This review summarizes the main molecular mechanisms through which the sex hormones such as androgens and estrogens and their corresponding receptors play very different roles in the progression of an HBV infection and in the development of HBV-related HCC between men and women.

## 2. Different Roles of Sex Hormones in HBV-Associated Hepatocarcinogenesis

As mentioned above, HBV-associated HCC tends to be higher in males and postmenopausal females than other females [22, 24–26]. Historically, scant attention has been given to the reasons for this difference in susceptibility, but in the past decade several studies demonstrated that sex hormones such as androgen (A) and estrogen play very different roles in the progression of an HBV infection and in the development of HBV-related HCC [27]. For instance, it was shown that female HBV carriers generally have lower viral loads than male carriers [28–30]. The same authors also reported that the ratio between estradiol and testosterone levels tends to decrease in female patients with HBV-associated HCC rather than in female HBV carriers who do not develop HCC. Notably, pregnancy, which markedly increases serum estrogen levels, was also reported to exert a protective effect against HCC and the protection enhanced with the number of full-term pregnancies (FTP) [31]. Other studies also suggested that higher levels of androgen signaling, reflected by higher testosterone levels in serum, may be associated with an increased risk of HBV-related HCC in men [32]. Even when the condition of their alcoholic cirrhosis or viral hepatitis improved, their risk for HCC was still higher [28, 32]. The chronic HBV carrier prevalence was also observed to be higher in males than in females (10.7% versus 4.4%) with vaccination at birth and follow-up for over 18 years [33]. In addition, substituted androgens have been associated with development of HCC in patients with aplastic anemia [34] and Fanconi's anemia [35]. Similar results were also observed in HBV transgenic animal models. For example, Farza and colleagues [36] performed one of the seminal studies to analyze the expression of hepatitis B surface antigen (HBsAg). Specifically, they reported that HBsAg mRNA level and serum HBsAg at different stages of development were higher in adult male mice compared to female mice and were dependent on sex hormones regulation. It was also observed that, after testis castration, a decrease of HBsAg gene expression could be immediately recorded, but this phenomenon was easily reversed by providing androgens through an exogenous source. Further studies in a model of HBV transgenic mice confirmed the role of androgens in maintaining higher serum HBsAg level in adult male mice [37]. The demonstration that higher serum levels of HBsAg in transgenic male mice was an androgen upregulating effect was finally provided by Breidbart et al. [38]. A massive reduction of the androgen receptor (AR) activity was also discovered in mice carrying mutant Tfm allele of AR where HBsAg levels are strongly reduced. These findings collectively indicate that A/AR signaling may promote HBV-related hepatic carcinogenesis, at least in the early stages, and that estrogen signaling might suppress hepatocarcinogenesis [39].

## 3. Potential Mechanisms by Which Androgen and Its Receptor Stimulate HBV Transcription and Promote HBV-Associated HCC

In adult stages, sex hormones such as androgens and estrogens are molecules derived from cholesterol and mainly synthesized in the testes and ovaries, respectively. Once produced, these gonad-derived hormones are released into the systemic circulation to exert their biological activity in responsive tissues [40–42]. Sex hormones are small lipophilic ligands that easily diffuse across the plasma membrane and directly bind to their cognate inactive nuclear receptors, AR or estrogen receptor (ER), within target cells. Specifically, AR exerts physiological and pathological functions in target tissues by translocating into the nucleus upon binding to androgens [40, 43] where it binds to specific DNA sequences known as androgen response elements (AREs) [40, 44] in conjunction with several AR cofactors [45]. The AR complex can therefore modulate the expression of genes that are involved in various physiological functions or pathological scenarios. The effects of androgen and its receptor can be either transitory or long term and can have either local or systemic impacts on organ function [46]. Studies by Nakatani et al. [47] reported that the change in exogenous and endogenous androgens level in male mice might lead to altered HCC progression. The authors have demonstrated that liver tumor is testosterone-responsive and hormonal manipulation by chemical orchidectomy could markedly reduce the appearance of liver tumors in male mice. Using mice lacking AR in hepatocytes, Ma et al. [48] also revealed that AR, but not androgens, plays a primary role in hepatocarcinogenesis. In fact, they also found that the incidence of carcinogen-induced hepatocarcinogenesis was lower in mice lacking AR in hepatocytes compared to wild-type mice, although the serum testosterone levels in both showed little difference [48]. The liver is the largest visceral organ responsible for systemic homeostasis of blood glucose as well as lipid and protein metabolism [49]. Recently, it has been considered as a sexually dimorphic organ, which expresses both AR and ER-*α*, and thus is responsive to sex hormones for the disparity reported in gene expression pattern, xenobiotic metabolism, and immune responses between men and women. [50–52]. The male predominance in HCC suggests that A/AR may promote and that estrogens/ERs may suppress hepatocarcinogenesis [39]. In fact, the AR, a ligand-dependent transcription factor of the nuclear receptor superfamily, was reported to be frequently overexpressed in HCC [53]. Wang et al. [54] instead provided an elegant demonstration of the HBx viral protein involvement. Specifically, they suggested that the androgen signal can stimulate the transcription and replication of HBV genes by directly binding to the cognate ARE within HBV enhancer I (Enh I) of the HBV genome, leading to HBx expression. Once produced, HBx activates c-Src kinase and inactivates glycogen synthase kinase (GSK-3) contributing to full activation of AR [55–57]. Disruption of these two ARE sites was able to suppress AR-induced HBV mRNA levels increase, suggesting that these cis-elements were the major sites responsible for the transcriptional modulation of HBV by the hepatic A/AR pathway. Similar studies by Wu et al. [53] also described a lower incidence of HBV-induced HCC among male mice with a dysfunction of hepatic AR compared to wild-type (WT) mice. Upregulation of the HBx antigen was shown to be a direct consequence of the binding to the ARE sites within HBV Enh I; therefore AR is important to increase HBV replication in HBV transgenic mice. In addition, AR-induced HBx expression is able to enhance AR transactivation; therefore an amplification of its role during hepatic cell transformation is warranted. The results collectively indicate that HBx and the androgen signaling pathway may establish a positive cross-talk during HCC development in chronically infected male carriers and clinical reports on male HBV patients seem to reinforce this hypothesis [20]. Recently, Feng and colleagues [58] also identified another important self-amplifying positive regulatory circuit including AR, cell cycle-related kinase (CCRK), and *β*-catenin. Specifically, they suggested that the androgen signaling pathway could stimulate transcription and cell cycle-related kinase (CCRK) expression by directly binding to ARE of the CCRK promoter region in HCC cell lines and primary HCC specimens from patients with HBV infection. Subsequently, both stimulation of AR expression and cell cycle progression were shown to be greatly promoted by activating the *β*-catenin/T cell factor (TCF) signaling pathway which follows an increased expression of CCRK in immortalized human liver cells. Further studies also indicate that CCRK might contribute to HCC development and progression by upregulation of epidermal growth factor receptor (EGFR), an effector of the mitogenic signal capable of stimulating cell proliferation in mouse liver [59] and angiogenesis in human HCCs [60, 61]. In summary, these results suggest that a self-amplifying positive regulatory loop including AR, CCRK, and *β*-catenin might play an important role in HBV-associated hepatocarcinogenesis [58]. In addition, they also indicate that the AR-CCRK-*β*-catenin-positive regulatory circuit might be a target of intense interest to elaborate novel therapeutic approaches in future, especially in male-predominant malignant tumors such as HBV-related HCC.

## 4. The Negative Role of Estrogen Signal in HBV Transcription and Protection against the Development of HBV-Associated HCC

The estrogen pathway has long been considered as a tumor protector against the progression of HBV infections and development of HBV-associated HCC [24, 54]. Studies performed by Almog et al. [62] in fact demonstrated that the estrogen signal was capable of diminishing the level of HBV mRNA in immunocompromised male mice transplanted with HBV transfected HepG-2 cells. Moreover, Maria and colleagues [63] have also identified a variant of ER expressed in men and postmenopausal women with HCC. However, other studies also showed that females living with HBV have a lower risk of developing HCC, when exposed to the use of oral contraceptives or hormone replacement therapy after menopause [54, 64]. Several mechanisms have been proposed to explain how estrogens inhibit HBV replication. For instance, it seems that estrogen signal can exert important roles in many biological processes primarily acting through two nuclear receptors, estrogen receptors *α* and *β* (ER-*α* and ER-*β*) [24, 65]. While the AR causes the increase of HBV RNA transcription, estrogen can increase the hepatic expression of its nuclear receptor ER-*α* to decrease HBV RNA transcription by inhibiting the regulatory function of viral Enh I present in the HBV genome. At the molecular level, ER-*α* plays an important role in reducing HBV transcription by physically interacting with the hepatocyte nuclear factor 4*α* (HNF-4*α*) and by blocking the HNF-4*α* binding to Enh I region of the HBV genome [64, 66]. Consistent with this view, most of the ER-*α*-mediated repressive activity was inhibited by disrupting the HNF-4*α* binding site located in the HBV genome [64]. Another important aspect of estrogen signal which is different from the AR is that some of the hepatic ER-*α*'s are localized in the nucleus and then they can also function alone in the absence of estrogen [64]. Other studies by Han et al., [67] also indicate that HBx can inhibit ER-*α* transcriptional activity by forming a ternary complex with ER-*α* and histone deacetylase 1 (HDAC1). This class of enzymes catalyzes deacetylation of hyperacetylated histone tails by cleavage of acetyl groups, leading to a compact chromatin structure that represses gene expression [16]. From these serial studies, it appears clearly that sex hormones exert an opposite effect on viral replication and protein expression differently influencing the progression of HBV infection and HBV-associated HCC development.

## 5. Gender Disparity in the Pattern of Expression of Specific miRNAs and Inflammatory Cytokines and Development of HBV-Related HCC

Increasing evidence suggests that HCC develops through a multistep tumorigenic process, affecting at the molecular level several carcinogenic-related genes by genetic and/or epigenetic alterations [23]. It is well documented that many of the HCC risk factors involve epigenetic alterations such as DNA methylation, histone modifications, and noncoding miRNAs [20–23]. Specifically, miRNAs are a family of small noncoding RNAs of 20–22 nucleotides that typically inhibit target gene expression by binding to the 3′untranslated region (3′-UTR) of target mRNA, resulting in either mRNA degradation or inhibition of translation, and have major roles in cellular function [68, 69]. MiRNA levels are deregulated in many diseases. In recent years, miRNAs have been found to be differentially expressed in several HCC tissues compared to corresponding adjacent nontumorous tissues [4, 21, 70] either acting as oncogenic miRNAs (oncomirs) or tumor suppressor miRNAs (TS-miRNAs) [71, 72]. This observation strongly indicates that miRNA deregulation is a hallmark of liver carcinogenesis. It has also been reported that aberrant miRNA expression could be detected in chronic hepatitis and cirrhotic liver [70]. For example, Gao et al. [73] demonstrated that miRNA dysregulation was an early event involved in the various steps of hepatic carcinogenesis process. Specifically, these authors have shown the abnormal expression pattern of miRNA-224, miRNA-145, and miRNA-199b in premalignant liver tissues, advancing the hypothesis that they are the starters of the HBV-related HCC. Among these, miRNA-145 was significantly downregulated in precancerous low grade dysplastic nodules (LGDNs) and various human HCC cell lines. In addition, miRNA-145 overexpression suppressed cell proliferation and inhibited cell migration and invasion in both HepG2 and Hep3B cells. Therefore, these results collectively suggest that mi-RNA-145 may act as a negative regulator of proliferation and invasion of HCC cells and may be a candidate tumor suppressor miRNA that plays a crucial role in HCC development (Table 1) [73–75]. Accumulating evidence that sex hormones can consistently contribute to carcinogenesis process through modulating specific miRNAs has been documented in malignant tumors such as breast cancer and prostate cancer [76, 77]. Chen et al. [20] also showed a gender disparity in the pattern of expression of miRNA-216a in the precancerous hepatic tissue, demonstrating a preferential increase in males as well as its correlation with patient prognosis. The same authors also reported the potential mechanism by which pri-miRNA-216a could be activated transcriptionally by the androgen signal in a ligand-dependent manner and was further increased by the HBx viral protein (Table 1) [20]. As reported by Feng et al., [58] the CCRK activated by the androgen signal significantly contributes to male hepatocarcinogenesis. Therefore, it seems that an important positive regulatory loop exists between the HBx viral protein and the androgen signal in male HBV patients [20, 54, 55, 58, 78] that plays an important role in the early stage of hepatocarcinogenesis of HBV-related male HCC [20, 54, 55, 58, 78]. Increasing evidence also suggests that the effects of chronic inflammation and inflammatory cytokines such as interleukin-6 (IL-6) and interleukin-1*β* (IL-1*β*) may differently influence susceptibility to an HBV infection, persistence of an HBV infection, and the development of HBV-related HCC in male and female patients [17–19]. For instance, Naugler et al. [79] demonstrated that estrogen signal suppressed IL-6 production by Kupffer cells (KCs), resulting in reduced HCC incidence in females. The same authors also revealed that the estrogen signal inhibited IL-6 expression in KCs, resulting in reduced HCC incidence in the diethylnitrosamine (DEN) mouse model. The potential mechanism underlying this gender disparity suggests that estrogen and its corresponding receptor can reduce the IL-6 level and subsequent liver injury by reducing the activity of transcription factors such as nuclear factor-kappa B (NF-*κ*B), signal transducer and activator of transcription 3 (STAT3), CCAAT/enhancer binding protein *β* (C/EBP*β*), and the toll-like receptor adaptor protein MyD88 [79, 80]. Recent studies by Jiang et al. [17] also suggested that IL-1 can significantly contribute to development of HBV-related male HCC and that it can serve as an excellent biomarker for the determination of chronic liver inflammation and HBV-related HCC. Further studies by the same authors also reported that the estrogen signal was markedly reduced in both tumor and tumor adjacent tissues of males. Conversely, in some female patients, the estrogen signal was strongly expressed in both HCC and tumor adjacent tissue. In addition, IL-1*α* secretion was markedly suppressed by estradiol in necrotic HCC cell lines that showed increased expression of ER-*α*. These results collectively indicate that the protective anti-inflammatory effect induced by the estrogen signal was absent in male HCC tumors [17, 19]. One possible mechanism underlying this sex disparity suggests that in female the estrogen signal might block IL-1*α* transcription by interacting with the ER-*α* binding site of the human IL-1*α* promoter. In addition, in female miRNA-22 expression was not significantly increased. Conversely, in male, miRNA-22 expression was significantly increased in tumor adjacent tissue compared with normal tissue and downregulated in HCC tissues (Table 1) [17, 19, 81]. Two important reports by Liu et al. [82, 83] also revealed that in women miRNA-18a overexpression reduced the protective effect of the estrogen signal but increased the proliferation of hepatoma cells. This event was mediated by a mechanism probably involving tumor suppressor protein p53 (Table 1). Increasing evidence finally suggests that HBx may induce not only genetic modifications but also epigenetic changes, including aberrations in DNA methylation, histone modifications, and miRNA expression [23]. HBx can manipulate epigenetic mechanisms to modulate not only host gene expression in hepatocytes but also the replicative activity of HBV itself [84]. These results collectively suggest that sex disparity may play an important role in the progression of an HBV infection and in the initiation and progression of HBV-related HCC depending on the levels of sex hormones and inflammatory cytokines as well as on epigenetic changes in hepatic tissues such as aberrant miRNA expression, DNA methylation, and histone modifications related to HBx.

## 6. Conclusion

Several epidemiologic studies reported that HBV-related HCC is more frequent in males, while among females the incidence increases in postmenopausal period [5, 85]. Emerging experimental studies clearly suggest that sex hormones such as androgens and estrogens and their corresponding receptors (AR and ER-*α*) play very different roles in the HBV infection process and in HBV-related HCC. Estrogens exert a plethora of protective effects; in this scenario they are able to reduce viral RNA transcription and HBx viral protein expression and regulate viral activity by directly inhibiting HNF-4*α* from binding to Enh I of the HBV genome. Moreover, the estrogen signal can also modulate the production of inflammatory cytokines (IL-1 and IL-6) reducing HCC incidence in females. The estrogen signaling pathway also exerts protective effects not affecting the expression of miRNA-216a in precancerous liver tissues of female. Another important aspect of estrogen signal is that, in female HCC patients, increased expression of miRNA-18a due to elevated/mutant p53 suppresses the protective effect of estrogen by reducing ER-*α* levels via binding to its mRNA. This can significantly contribute to the development of HBV-related hepatocarcinogenesis process. Notably, miRNAs are key regulators of HBV-related HCC. Indeed, in men, the androgen signal can significantly increase the risk of HBV-related HCC development by increasing miRNA-22 expression in tumor adjacent tissue. Once produced, miRNA-22 might suppress ER-*α* transcription by reducing the protective effect of estrogen and causing increased IL-1*α* expression that acts as a crucial growth factor in HBV-associated hepatocarcinogenesis. Interestingly, the androgen/AR pathway is also involved in miRNA-216a upregulation in precancerous liver tissue of male HBV-related HCC. Notably, since such patients have a higher risk of HBV-related HCC development, miRNA-216a levels can also be used as excellent prognostic biomarkers. Finally, in male patients, the androgen signal can also stimulate the HBV RNA transcription and HBx viral protein expression by binding to ARE sites in enhancer I of the HBV genome. HBx can indirectly increase the AR mRNA levels by inactivating GSK-3 and activating c-Src kinase contributing to full activation of AR. HBx protein was also investigated for the property of promoting HBV-related hepatocarcinogenesis by altering normal physiologic mechanisms of the host cell [23]. For instance, genetic and epigenetic alterations related to HBx protein may associate with gender difference and play a key role in the development and progression of HBV-related HCC, but the exact mechanisms by which HBx protein disrupts these genetic and epigenetic changes are still not well known and require deeper investigations [23]. In addition, certain epigenetic alterations can also be used either as excellent biomarkers for diagnosis, prognosis, and monitoring or as excellent targets for epigenetic therapy [23]. Despite the extensive research done in this subject area, very few studies have examined the influence of HBx viral protein in the initiation of HBV-related HCC through the modulation of NF-E2-related factor (Nrf2) transcription factor [86, 87] and clock genes [88]. Notably, Nrf2 is the master regulator involved in the induction of several antioxidant and phase II detoxification enzymes by short cis-acting elements in their promoters, called antioxidant response elements (AREs). Therefore, its HBx inhibition might be related to the initiation of HBV-associated HCC. Moreover, during chronic infection, the capacity of HBV to induce the expression of augmenter of liver regeneration (ALR) gene and other Nrf2/ARE-regulated genes could also protect HBV-positive cells and thereby ensure viral replication and subsequent neoplastic transformation [86, 87]. Consequently, manipulating Nrf2 activity might be a useful strategy for treating HBV-associated liver carcinogenesis. Recent studies by Yang et al. [88] also suggest that HBx alters the circadian clock genes expression and can, therefore, promote the HCC development. The alteration of circadian clock gene expression has been found in various malignant tumors. Indeed, circadian clocks also modulate a variety of cancer-related genes, including genes that control cell-cycle progression (e.g., c-Myc, cyclin D1, cyclin A, p53, and Mdm2), differentiation, DNA repair, and apoptosis (e.g., caspases) [88, 89]. Therefore, the dysregulation of circadian clock genes and their downstream clock-controlled genes can have a profound influence on HCC development [88, 89]. Until now we have to address the idea that there are no effective therapeutic strategies for HCC [26, 90]. Tamoxifen, known to be an estrogenic antagonist used for estrogen positive breast cancer, showed no significant beneficial effect [26, 91–94]; in the same way, flutamide has not registered a remarkable advantage [95]. To date, since there is no information on the mechanisms underlying gender differences associated with HBV-related HCC, there is a major limitation to the use of innovative and effective treatments in advanced hepatocellular carcinoma. Further studies are needed to explore this field still largely obscure.

## Figures and Tables

**Table 1:** Functional activities of specific miRNAs in HBV-associated hepatocarcinogenesis process between female and male.

miRNA-145	In HBV-related HCC patients the global miRNA expression profiles are different from those of nontumorous liver tissues. Among examined miRNAs, miRNA-145 is frequently downregulated in HBV-associated HCC patients. Its overexpression can inhibit cell proliferation and invasion of HCC cells. Therefore, it has been suggested that miRNA-145 can act as a negative regulator of proliferation and invasion of HCC cells. In addition, it also regulates some properties of cancer stem cells (CSCs) such as stem cell differentiation and stem cell self-renewal by negatively modulating the expression of protooncogene c-myc and pluripotency factors SOX-2, OCT4, and KLF4. These data suggest that miRNA-145 can be a tumor suppressor miRNA and may play a crucial role in HCC development

miRNA-216a	Various studies showed that there is a gender disparity in the pattern of expression of miRNA-216a in precancerous liver tissues. In female no upregulation of miRNA-216a expression was observed. Conversely, in premalignant liver tissues of male HBV-related HCC patients, the androgen signal can activate and increase miRNA-216a transcription by directly binding to the site ARE within the promoter region of pri-miRNA-216a. This leads to an increase of the miRNA-216a level and subsequent suppression of its target genes (e.g., TSCL1) through an important self-amplifying positive loop including AR, CCRK, and *β*-catenin/T cell factor (TCF) signaling in HCC cell lines and animal models. Notably, miRNA-216a levels can also be used as excellent prognostic biomarkers. In addition, ligand-activated AR can also stimulate the HBV RNA transcription by binding to ARE sites in enhancer I of the HBV genome. This leads to replication of HBV genes and HBX expression which in turn activates c-Src kinase and inactivates GSK-3 contributing to full activation of AR. These results indicate that there is an interesting positive cross-regulatory loop between the androgen signal and the HBX viral protein that can significantly contribute to male HBV-related HCC development

miRNA-22	In some female patients, the estrogen signal exerts oncosuppressive activity by suppressing IL-1*α* transcription by binding to the human IL-1*α* promoter. In addition, in female miRNA-22 expression is not significantly increased. Conversely, in male the androgen signal exerts oncogenic activity by increasing miRNA-22 expression in tumor adjacent tissue compared with controls. Increased expression of miRNA-22 in male tumor adjacent tissue might inhibit ER-*α* expression by directly targeting the 3′-UTR region of ER mRNA. This leads to attenuation of the protective effect of estrogen, causing increased IL-1*α* expression. Finally, this persistent overexpression of IL-1*α* from necrotic hepatocytes might lead to a compensatory proliferative response and tumorigenesis in normal hepatic cells.

miRNA-18a	In female, increased expression of miRNA-18a due to elevated/mutant p53 reduces ER-*α* levels by binding to its mRNA at the 3′-untranslated region (3′-UTR). It seems that elevated/mutant p53 plays a key role in modulating the amount of ER-*α* protein by promoting the biogenesis of miRNA-18a. By this mechanism, miRNA-18a could suppress the estrogen signal and promote the development of HCC in women.
